# Prevalence and Childhood Precursors of Opioid Use in the Early Decades of Life

**DOI:** 10.1001/jamapediatrics.2020.5205

**Published:** 2020-12-28

**Authors:** Lilly Shanahan, Sherika N. Hill, Laura Bechtiger, Annekatrin Steinhoff, Jennifer Godwin, Lauren M. Gaydosh, Kathleen Mullan Harris, Kenneth A. Dodge, William E. Copeland

**Affiliations:** 1 Jacobs Center for Productive Youth Development, University of Zurich, Zurich, Switzerland; 2 Department of Psychology, University of Zurich, Zurich, Switzerland; 3 Department of Psychiatry and Behavioral Sciences, Duke University Medical Center, Durham, North Carolina; 4 Center for Child and Family Policy, Duke University, Durham, North Carolina; 5 Center for Medicine, Health, and Society, Public Policy Studies, Vanderbilt University, Nashville, Tennessee; 6 Carolina Population Center, Department of Sociology, University of North Carolina at Chapel Hill; 7 Department of Psychology and Neuroscience, Duke University, Durham, North Carolina; 8 Vermont Center for Children, Youth, and Families, Department of Psychiatry, University of Vermont, Burlington

## Abstract

**Importance:**

Opioid use disorder and opioid deaths have increased dramatically in young adults in the US, but the age-related course or precursors to opioid use among young people are not fully understood.

**Objective:**

To document age-related changes in opioid use and study the childhood antecedents of opioid use by age 30 years in 6 domains of childhood risk: sociodemographic characteristics; school or peer problems; parental mental illness, drug problems, or legal involvement; substance use; psychiatric illness; and physical health.

**Design, Setting, and Participants:**

This community-representative prospective longitudinal cohort study assessed 1252 non-Hispanic White individuals and American Indian individuals in rural counties in the central Appalachia region of North Carolina from January 1993 to December 2015. Data were analyzed from January 2019 to January 2020.

**Exposures:**

Between ages 9 and 16 years, participants and their parents were interviewed up to 7 times using the Child and Adolescent Psychiatric Assessment and reported risk factors in 6 risk domains.

**Main Outcomes and Measures:**

Participants were assessed again at ages 19, 21, 25, and 30 years for nonheroin opioid use (any and weekly) and heroin use using the structured Young Adult Psychiatric Assessment.

**Results:**

Of 1252 participants, 342 (27%) were American Indian. By age 30 years, 322 participants had used a nonheroin opioid (24.2%; 95% CI, 21.8-26.5), 155 had used a nonheroin opioid weekly (8.8%; 95% CI, 7.2-10.3), and 95 had used heroin (6.6%; 95% CI, 5.2-7.9). Childhood risk markers for later opioid use included male sex, tobacco use, depression, conduct disorder, cannabis use, having peers exhibiting social deviance, parents with legal involvement, and elevated systemic inflammation. In final models, childhood tobacco use, depression, and cannabis use were most robustly associated with opioid use in young adulthood (ages 19 to 30 years). Chronic depression and dysthymia were strongly associated with any nonheroin opioid use (OR. 5.43; 95% CI, 2.35-12.55 and OR, 7.13; 95% CI, 1.99-25.60, respectively) and with weekly nonheroin opioid use (OR, 8.89; 95% CI, 3.61-21.93 and OR, 11.51; 95% CI, 3.05-42.72, respectively). Among young adults with opioid use, those with heroin use had the highest rates of childhood psychiatric disorders and comorbidities.

**Conclusions and Relevance:**

Childhood tobacco use and chronic depression may be associated with impaired reward system functioning, which may increase young adults’ vulnerability to opioid-associated euphoria. Preventing and treating early substance use and childhood mental illness may help prevent later opioid use.

## Key Points

**Question:**

How common is opioid use in the early decades of life, and which childhood risk factors are associated with opioid use in young adulthood?

**Findings:**

This cohort study assessed opioid use among 1252 non-Hispanic White individuals and American Indian individuals in rural counties in the central Appalachia region of North Carolina from January 1993 to December 2015. By age 30 years, approximately one-quarter of participants had used opioids, and the findings revealed that childhood tobacco use and depression were associated with later nonheroin opioid use in general, weekly nonheroin opioid use, and heroin use.

**Meaning:**

Childhood tobacco use and depression may be associated with impaired reward system functioning, which may increase young adults’ vulnerability to opioid-associated euphoria.

## Teaser

This cohort study documents age-related changes in opioid use and analyzes childhood antecedents of opioid use among non-Hispanic White individuals and American Indian individuals.

## Introduction

Beginning in the late 1990s, when opioids were prescribed with few restrictions, opioid use in the US rose to epidemic levels.^1^ Prescription practices, which made these highly addictive drugs easily accessible through medical and nonmedical channels,^2,3,4,5^ have been overhauled,^6,7^ but despite some progress in addressing the opioid epidemic, it remains unclear how young adults became part of this epidemic. Young adults typically do not experience age-related pain problems that warrant opioid prescriptions, but their premature mortality from opioid overdoses has skyrocketed.^8,9,10,11^ This prospective longitudinal study measured childhood adversities in opioid-naive children from ages 9 to 16 years and examined the age-related course of opioid use and associations between childhood risk factors (ages 9 to 16 years) and opioid use in young adulthood (ages 19 to 30 years).

Opioid use among young people in the US has been documented by the Monitoring the Future study,^12,13,14^ the National Survey on Drug Use and Health,^9,10,15,16,17,18^ and the National Epidemiological Survey on Alcohol and Related Conditions,^19^ among other studies. These studies reported that a considerable percentage of young people have used opioids^13^; the prevalence of opioid use is found to be higher among White adolescents than among Black and Hispanic adolescents in many, but not all, studies^12,14,15^; and sex differences in opioid use are inconsistent.^12^ Most of these studies are cross-sectional or short-term longitudinal studies and therefore cannot uncover how opioid use and differences by sex and race/ethnicity unfold from adolescence onward.

Several retrospective cross-sectional and prospective short-term longitudinal studies have identified childhood adversity,^14,15^ school problems,^14,20^ psychiatric problems,^12,14,15,16^ and early substance use^12,14,20^ as associated with later opioid use or misuse. In addition, medically relevant factors, including injuries, pain problems, and nonmedical use of prescription opioids, predicted later opioid use.^12,21,22,23^ To our knowledge, no previous study has examined associations between experiences assessed in childhood and opioid use in young adults. Most longitudinal analyses of opioid use begin in late adolescence (eg, age 18 years in the Monitoring the Future study),^14^ relying on retrospective assessments of childhood experiences, which may be affected by forgetting and recall bias.^24,25^

Our prospective longitudinal cohort study spanning 20 years examines the prevalence of any and weekly nonheroin opioid use and any heroin use from ages 9 to 30 years. It also tests which childhood risk factors are associated with later opioid use. Data came from White and American Indian participants in the central Appalachia region of North Carolina, an epicenter of the opioid crisis.^26^ American Indian individuals tend to be understudied^27,28^ and are considered at high risk of substance use problems in adolescence.^29,30^ They also experience high rates of premature mortality because of drug and alcohol use.^31^

## Methods

### Participants

This study drew on the Great Smoky Mountains Study (GSMS),^32^ which is a longitudinal representative study of children in 11 predominantly rural counties of North Carolina.^32^ Three cohorts, aged 9, 11, and 13 years, were recruited from a pool of approximately 12 000 children using a 2-stage sampling design (eFigure 1 in the Supplement), resulting in 1420 participants (49% female).^32^ Potential participants were randomly selected from the population using a household equal probability design and screened for risk of psychopathology; those scoring high for risk of psychopathology were oversampled and the rest were randomly sampled. American Indian children were oversampled to constitute 25% of the total sample.^32,33,34^ The Duke University Medical Center Institutional Review Board approved the study, and participants and their parents or guardians signed informed consent forms. Participants were paid $20 to $100. This report followed the Strengthening the Reporting of Observational Studies in Epidemiology (STROBE) reporting guideline.

Participants and a parent figure (typically the mother) completed an annual assessment from ages 9 to 16 years (January 1993 to December 2000). Only participants responded at ages 19, 21, 25, and 30 years (January 1999 to December 2015). Analyses focused on White and American Indian participants with 1 or more young adult assessments.

### Assessment

The Child and Adolescent Psychiatric Assessment (CAPA) was used until age 16 years and the Young Adult Psychiatric Assessment (YAPA) thereafter.^35,36,37,38^ These structured interviews were coded by trained interviewers and checked by supervisors. In addition to opioid use and childhood risk factors, the interviews assessed sex and race/ethnicity. Race/ethnicity coding was based on parent-reported data collected at the first observation. Options were taken from the US Census. Race/ethnicity data were collected to study health disparities.

Lifetime nonheroin opioid and heroin use was assessed at each interview beginning at age 9 years using the CAPA/YAPA substance use module (2-week test-retest reliability, 0.98).^39^ Lifetime nonheroin opioid use was assessed by the question, “Have you tried any other opioids, like morphine, codeine, or other painkillers?” and questions about weekly use (“Have you used … at least once a week for a month or more?”). At age 30 years, a question about oxycodone use was incorporated into the any nonheroin opioid use variable. Nonheroin opioid use was assessed in the part of the interview on illegal substances; therefore, it is likely that our assessment included primarily nonmedical use. Medical use was not assessed separately. Lifetime heroin use was assessed with the question, “Have you ever tried heroin?” Binary opioid use variables were coded as 1 for use and 0 for no use for any nonheroin opioid use, weekly nonheroin opioid use, and any heroin use. These outcome variables were not mutually exclusive.

We selected childhood risk factors known to be associated with opioid use or substance use generally, including sociodemographic risk or family dysfunction; school or peer problems; parental mental illness, drug problems, or legal involvement; early substance use; and physical health risks (Table 1). Physical health risks included systemic inflammation (assessed by C-reactive protein [CRP] level^40^), which is an objective biomarker associated with chronic pain and somatic symptoms^41^ as well as depression.^42^ In addition, CAPA interviews assessed child symptoms of psychiatric disorders. Child and parent reports were typically combined using an either/or rule to code children’s *Diagnostic and Statistical Manual of Mental Disorders* (Fourth Edition) diagnoses at each assessment. The 2-week test-retest reliability of CAPA diagnoses is comparable with other highly structured child psychiatric interviews.^39,43^ The recall time frame for childhood psychiatric status and risk factors was generally the previous 3 months.^39,43^

**Table 1.** Definitions and Assessments of Childhood Risk Factors

Risk factor	Definition/assessment, coded if criteria were met during ≥1 childhood assessment^a^
Sociodemographic risk or family dysfunction	
Family low socioeconomic status	Child’s family met ≥2 of the following conditions: below the federal poverty line for family income, parental high school education only, low parental occupational prestige
Family instability	Child’s family met ≥2 of the following conditions: single-parent structure, stepparent in household, divorce, parental separation, change in parent structure
Family dysfunction	Child’s family met ≥5 of the following: lax parental supervision, parental overinvolvement, physical violence between parents, frequent parental arguments, parental apathy, involvement of the child in parental arguments, current maternal depression, high conflict between child and parent, parental activities being a source of tension or worry for the child
Maltreatment	Lifetime physical or sexual abuse or current neglect (neglect was assessed by interviewers only)
Child’s school or peer problems	
Expelled from school	Child was suspended or expelled from school
Peers exhibiting social deviance	Child’s friends are often in trouble, disruptive of others, disrespectful to adults, drink alcohol, steal, or engage in other socially deviant behaviors (assessed during the first 3 waves of data collection only)
Mostly older friends	Most of child’s friends are ≥2 y older than the child
Experienced bullying	Child was bullied (eg, mocked, attacked, threatened) by peers at school, by siblings at home, or in other settings
Parents’ mental illness, drug problems, legal involvement	
Parental mental health service use	≥1 Parent had used services for mental health problems during their lifetime (eg, sought or received treatment, hospitalization, medication)
Parental drug service use	≥1 Parent had used services for drug or alcohol problems during their lifetime (eg, sought or received treatment, hospitalization)
Parent with legal involvement	≥1 Parent had been arrested or prosecuted during their adult life
Child’s early substance use	
Tobacco use	Any tobacco use
Alcohol use	Any alcohol use
Cannabis use	Any cannabis use
Illicit drug use other than cannabis	Any illicit drug use other than cannabis or opioid use
Child’s psychiatric risks (*DSM-IV* diagnoses)	
Anxiety disorder	Child met *DSM-IV* diagnostic criteria for ≥1 anxiety disorder: generalized anxiety disorder, overanxious disorder, social phobia, separation anxiety disorder, simple phobia, panic disorder, or agoraphobia
Depressive disorder	Child met *DSM-IV* diagnostic criteria for ≥1 depressive disorder: major depressive disorder, dysthymia, or minor depression (depression not otherwise specified)
Oppositional defiant disorder	Child met *DSM-IV* diagnostic criteria for oppositional defiant disorder
Conduct disorder	Child met *DSM-IV* diagnostic criteria for conduct disorder
Attention-deficit/hyperactivity disorder	Child met *DSM-IV* diagnostic criteria for attention deficit hyperactivity disorder (assessed from parent report only)
Psychiatric comorbidity	Child met criteria for ≥2 *DSM-IV* psychiatric disorders simultaneously
Child’s physical health	
Obesity	Child’s body mass index was calculated from weight and height at each assessment; obesity was coded when the child met US Centers for Disease Control and Prevention criteria for obesity
Somatic complaints	Child experienced frequent and recurrent (ie, at least weekly) headaches, abdominal, or muscular/joint pain over a minimum of a 3-mo period
Injury	Child experienced a physical injury in the past year (assessed from parent report only)
Elevated systemic inflammation (high C-reactive protein level)	C-reactive protein level ≥3 mg/L; data derived from blood spots that were obtained during each childhood interview

Abbreviation: *DSM-IV*, *Diagnostic and Statistical Manual of Mental Disorders* (Fourth Edition).

^a^ To maximize recall reliability, the typical recall time frame used in Great Smoky Mountains Study assessments was the previous 3 months at each assessment.

### Statistical Analysis

Prevalence estimates were weighted with sampling weights to adjust for differential probability of selection and to generalize results to the broader population from which the sample was drawn. Numbers of observations reported were unweighted. Childhood associations with adult opioid use were tested using weighted logistic regression analyses in SAS/STAT software version 9.4 (IBM). In step 1, childhood risk factors were entered into models separately, adjusting for control variables (sex, race/ethnicity, cohort). Product terms between each risk factor and sex and race/ethnicity, respectively, tested whether associations varied by sex or race/ethnicity. In step 2, control variables and risk factors from a given risk domain were entered into models simultaneously. In step 3, control variables and all significant associations from step 2 were entered simultaneously, trimming all associations with *P* ≥ .10. Sandwich-type variance corrections^44^ were applied to adjust for parameter and variance effects induced by sampling stratification. *P* values were 2-tailed, and significance was set at *P* < .05. In addition, we examined odds ratios (ORs) with a size of 2 or more. Attrition was low: 1336 GSMS participants (94.1%) provided at least 1 young adult interview. Data were analyzed from January 2019 to January 2020.

## Results

### Cumulative Lifetime Prevalence of Opioid Use From Childhood to Early Adulthood

There were a total of 1252 non-Black participants with observations in young adulthood, 342 (27%) of whom were American Indian. Although Black children participated in the GSMS, this subsample was too small (n = 88) for robust tests of race/ethnicity differences and was excluded. Notably, however, their lifetime prevalence of opioid use was low: 12.5% of Black participants reported any nonheroin opioid use by age 30 years. The Figure shows the cumulative lifetime estimates, derived from repeated lifetime assessments of opioid use from ages 9 to 30 years. By age 30 years, 322 participants had used a nonheroin opioid (24.2%; 95% CI, 21.8-26.5); 155 had used a nonheroin opioid weekly (8.8%; 95% CI, 7.2-10.3; 35.8% of those with any opioid use); and 95 had used heroin (6.6%; 95% CI, 5.2-7.9; 21.8% of those with any opioid use). The overlap among opioid use variables was significant: 78 participants (80.3%) with lifetime heroin use at age 30 years had used other opioids (52 [42.5%] weekly). In addition, 52 (31.9%) of those with weekly nonheroin opioid use had also used heroin.

**Figure.**
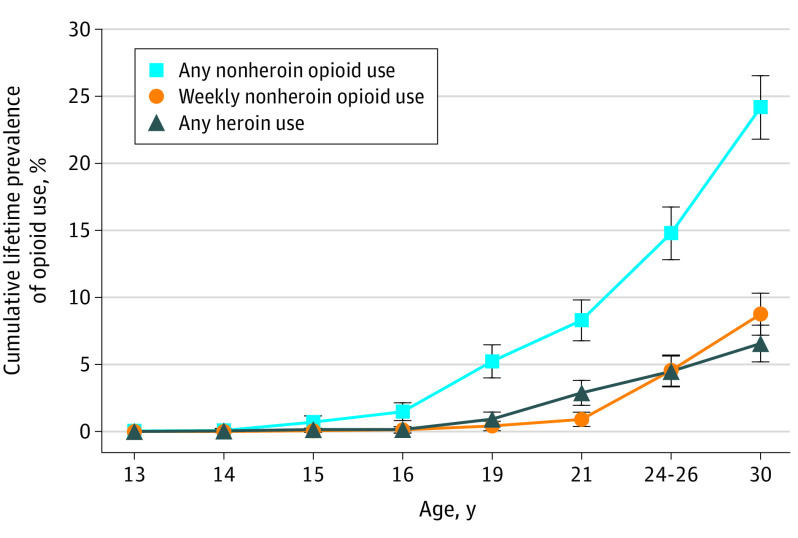
Cumulative Lifetime Prevalence of Opioid Use From Ages 13 to 30 Years Error bars indicate 95% CIs.

The prevalence of opioid use varied by race/ethnicity and sex (eFigures 2, 3, and 4 in the Supplement). By age 30 years, male individuals had a higher lifetime prevalence of any opioid use (214 [28.6%]; 95% CI, 25.1-32.1) and heroin use (70 [9.0%]; 95% CI, 6.7-11.2) than female individuals (108 [19.7%]; 95% CI, 16.6-22.8 and 25 [4.1%]; 95% CI, 2.6-5.7, respectively) (OR, 1.63; 95% CI, 1.05-2.53; *P* = .03; OR, 2.29; 95% CI, 1.0-5.17; *P* = .045 for sex differences, respectively). American Indian individuals reported higher weekly nonheroin opioid use than White individuals (104 [8.5%]; 95% CI, 11.3-19.1; 51 [14.9%]; 95% CI, 6.9-10.1; OR, 1.89; 95% CI, 1.22-2.93, *P* = .005).

### Childhood Risk Factors and Young Adult Opioid Use

The analytic sample was evenly divided by sex (677 [50.3%] male). The prevalence of childhood risk factors, aggregated from ages 9 to 16 years, was divided into groups according to no lifetime use of opioids, any nonheroin opioid use, weekly nonheroin opioid use, and heroin use (eTable 1 in the Supplement). The ORs of the association between each risk factor and opioid use by age 30 years, adjusted for control variables (step 1 of the analytic strategy), are shown in eTable 2 in the Supplement.

Table 2 shows associations among the risk factors and opioid outcomes when all risk factors within a risk domain were entered into a multivariate model simultaneously (step 2 of the analytic strategy). Childhood risk factors associated with at least 2 of 3 opioid outcomes included tobacco use, depressive disorders, conduct disorders, having peers exhibiting social deviance, parents with legal involvement, and elevated systemic inflammation (high CRP level). Several risk factors, including childhood tobacco use, depressive disorders, and conduct disorders, were associated with both nonheroin opioid use and heroin use. The associations did not meaningfully vary by sex or race/ethnicity (eTables 3 and 4 in the Supplement). The overall pattern of results held when participants who had used opioids by age 16 years were removed (eTable 5 in the Supplement).

**Table 2.** Results From Multivariate Models That Entered Risk Markers Within Each Risk Domain Simultaneously, Adjusting for Sex, Race/Ethnicity, and Cohort

Childhood risk factors	Prevalence of risk, No. (%)	OR (95% CI)		
		Any nonheroin opioid use	Weekly nonheroin opioid use	Any heroin use
Total, No.	1252	322	155	95
Sociodemographic and family characteristics				
Family low SES	491 (26.7)	0.93 (0.55-1.55)	1.13 (0.53-2.43)	0.73 (0.30-1.80)
Family instability	377 (24.2)	1.46 (0.88-2.42)	0.88 (0.46-1.70)	2.11 (0.86-5.20)
Family dysfunction	214 (13.6)	1.38 (0.76-2.53)	1.41 (0.67-2.96)	0.79 (0.31-2.01)
Maltreatment	503 (31.0)	1.23 (0.74-2.05)	1.46 (0.73-2.92)	1.79 (0.73-4.38)
Child’s school/peer risk				
Expelled from school	86 (4.5)	1.43 (0.61-3.36)	1.55 (0.56-4.31)	2.51 (0.86-7.33)^c^
Peers exhibit social deviance	528 (33.5)	2.38 (1.47-3.86)^a^	3.93 (2.06-7.51)^b^	1.46 (0.67-3.18)
Mostly older friends (≥2 y)	220 (12.3)	1.88 (1.06-3.34)^b^	0.92 (0.43-2.00)	1.13 (0.44-2.90)
Experienced bullying	432 (30.2)	1.47 (0.93-2.34)	1.57 (0.84-2.96)	1.81 (0.84-3.90)
Parental MI, drug, legal involvement				
Parental mental health service use	642 (48.5)	1.59 (0.98-2.57)^c^	0.80 (0.42-1.54)	1.88 (0.76-4.64)
Parental drug service use	299 (15.8)	0.86 (0.49-1.51)	1.32 (0.66-2.65)	2.30 (0.89-5.94)^c^
Parental legal involvement	661 (41.5)	1.91 (1.18-3.08)^d^	2.65 (1.33-5.29)^d^	0.68 (0.27-1.75)
Child’s substance use				
Tobacco	364 (22.3)	3.82 (2.17-6.72)^b^	6.64 (3.27-13.46)^b^	3.91 (1.56-9.83)^d^
Alcohol	285 (20.2)	1.32 (0.65-2.65)	1.13 (0.46-2.77)	1.41 (0.58-3.43)
Cannabis	208 (12.9)	2.84 (1.37-5.90)^d^	2.05 (0.78-5.41)	2.22 (0.85-5.78)
Other illicit drug	51 (2.6)	1.36 (0.32-5.83)	0.53 (0.21-1.36)	3.30 (0.89-12.26)^c^
Child’s psychiatric risk				
Anxiety disorders	176 (10.8)	1.11 (0.56-2.22)	0.81 (0.31-2.15)	1.34 (0.48-3.76)
Depressive disorders	128 (8.4)	2.63 (1.22-5.65)^a^	3.94 (1.49-10.39)^d^	6.32 (1.94-20.65)^b^
Oppositional defiant disorder	270 (13.5)	0.97 (0.51-1.85)	1.85 (0.69-4.93)	0.92 (0.37-2.26)
Conduct disorder	166 (8.1)	2.37 (1.27-4.44)^d^	2.25 (0.88-5.76)^c^	3.27 (1.32-8.10)^a^
ADHD	69 (3.5)	0.89 (0.32-2.49)	0.78 (0.26-2.31)	1.20 (0.31-4.57)
Comorbidity: ≥2 diagnoses^e^	247 (12.9)	NA	NA	NA
Child’s physical health				
Obesity	464 (28.6)	0.83 (0.50-1.38)	0.69 (0.34-1.39)	0.90 (0.45-1.80)
Somatic complaints	410 (31.2)	1.05 (0.66-1.68)	1.77 (0.97-3.21)^c^	2.55 (1.16-5.60)^a^
Injury	541 (42.4)	1.07 (0.68-1.67)	1.56 (0.83-2.91)	1.49 (0.69-3.22)
Elevated systemic inflammation (CRP level ≥3 mg/L)	312 (21.2)	1.87 (1.11-3.15)^a^	2.92 (1.44-5.92)^d^	1.91 (0.83-4.42)

Abbreviations: ADHD, attention-deficit/hyperactivity disorder; CRP, C-reactive protein; MI, mental illness; NA, not applicable; OR, odds ratio; SES, socioeconomic status.

^a^ *P* < .001.

^b^ *P* < .05.

^c^ *P* < .10.

^d^ *P* < .01.

^e^ Not included in multivariate models.

In the final models (step 3 of the analytic strategy), the following childhood risk factors for young adult opioid use emerged. For any nonheroin opioid use, risk factors included tobacco use (OR, 3.96; 95% CI, 2.28-6.53), cannabis use (OR, 3.28; 95% CI, 1.73-6.25), depression (OR, 1.82; 95% CI, 0.97-3.12), and male sex (OR, 1.52; 95% CI, 0.94-2.45); for weekly nonheroin opioid use, risk factors included tobacco use (OR, 5.89; 95% CI, 3.13-11.08), depression (OR, 2.59; 95% CI, 1.10-6.06), high CRP level (OR, 2.25; 95% CI, 1.13-4.48), and peers exhibiting social deviance (OR, 2.17; 95% CI, 1.16-4.04); for heroin use, risk factors included depression (OR, 5.54; 95% CI, 1.90-15.63), tobacco use (OR, 3.64; 95% CI, 1.46-9.09), cannabis use (OR, 2.82; 95% CI, 1.12-7.10), and male sex (OR, 2.53; 95% CI, 1.04-6.13).

### Association of Specific Childhood Depressive Symptoms and Diagnoses With Opioid Use

Although depression is a heterogeneous construct, eTable 6 in the Supplement shows that every depressive symptom, except motoric agitation or retardation and fatigue or lack of energy, was associated with weekly nonheroin opioid and any heroin use (OR near or above 2.0). Depressed or irritable mood, chronically low mood, worthlessness or guilt, and low self-esteem were associated with all opioid outcomes. Anhedonia, problems thinking or making decisions, suicidal ideation, and insomnia or hypersomnia were associated with both weekly nonheroin opioid use and any heroin use.

Table 3 shows associations of childhood major depressive disorder (MDD), minor depression, dysthymia, and chronic depression with young adult opioid use. By definition, dysthymia is more chronic than MDD or minor depression.^45^ Results suggest that childhood dysthymia and chronic depression are more strongly associated with later nonheroin opioid use (both weekly and any) than MDD or minor depression. Each childhood depressive disorder was strongly associated with heroin use.

**Table 3.** Associations Between Specific Childhood Depressive Disorders and Opioid Use by Age 30 Years^a^

Type of childhood depression	Prevalence, No. (%)	OR (95% CI)		
		Any nonheroin opioid use	Weekly nonheroin opioid use	Any heroin use
Total, No.	1252	322	155	95
Major depression	33 (2.3)	2.21 (0.76-6.44)	3.41 (0.92-12.59)^b^	7.56 (1.92-29.73)^c^
Minor depression	106 (7.4)	2.98 (1.43-6.22)^d^	4.46 (1.90-10.50)^d^	7.97 (3.18-19.67)^d^
Dysthymia	70 (5.2)	5.43 (2.35-12.55)^d^	8.89 (3.61-21.93)^d^	8.16 (2.96-22.46)^d^
Chronic depression (≥2 y of any type)	28 (2.4)	7.13 (1.99-25.60)^c^	11.41 (3.05-42.72)^d^	3.93 (0.77-20.06)^b^

Abbreviation: OR, odds ratio.

^a^ Independent predictors were adjusted for sex, race/ethnicity, and cohort.

^b^ *P* < .10.

^c^ *P* < .01.

^d^ *P* < .001.

### Putative Progression From Any to Weekly Heroin Use

Analyses comparing groups defined by different levels of opioid use are shown in eTables 7 and 8 in the Supplement. Specifically, we tested associations among childhood risk factors and weekly nonheroin opioid (vs any nonheroin opioid) use and heroin (vs weekly nonheroin opioid) use. These comparisons assume that those with weekly nonheroin opioid use progressed from any nonheroin opioid use and that those with heroin use progressed from weekly nonheroin opioid use. Putative progression to weekly nonheroin opioid use was associated with American Indian ethnicity, childhood tobacco use, psychiatric disorders, physical health problems, and having peers exhibiting social deviance. Putative progression from weekly nonheroin opioid use to heroin use was associated with childhood family instability, psychiatric disorders (eg, conduct disorder and attention-deficit/hyperactivity disorder), school or peer factors, alcohol use, and somatic complaints.

## Discussion

Our community-representative, prospective longitudinal study first assessed opioid-naive children aged 9 to 13 years. By age 30 years, 1 in 4 individuals (more male individuals than female individuals) living at the epicenter of the opioid epidemic had used nonheroin opioids. Childhood risk markers for later opioid use included male sex, tobacco use, depression, conduct disorder, cannabis use, having peers exhibiting social deviance, parents with legal involvement, and elevated systemic inflammation. In final models, childhood tobacco use and depression, particularly chronic depression, were among the key associations of young adult opioid use. Young adults with heroin use had complex mental health histories with the highest rates of childhood depression and psychiatric comorbidity. Putative progression from any to weekly nonheroin opioid use and then heroin use was associated with somewhat different sets of risk factors. Health factors and both depressive and conduct disorders were associated with progression from any to weekly use. Family instability, school or peer risk, and conduct disorder were associated with progression to heroin use.

### Childhood Depression and Later Opioid Use

Co-occurrence of lifetime MDD and adult problematic substance use^46,47,48,49,50^ and pathways from mood disorders to opioid dependence in adults have been documented.^51^ We add to these findings by showing that opioid-naive children experiencing depression are at increased risk of later opioid use. This is concerning considering that depressive symptoms among US children and adolescents have risen to their highest levels since 1991.^52^

One possible reason childhood chronic depression increases the risk of later opioid use is self-medication, including the use of psychoactive substances, to alleviate depression.^53,54,55,56^ Opioids may offer a problematic antidote to depression-related difficulties detecting and experiencing reward or pleasure, debilitating low moods, and low self-esteem. First-time use of opioids can induce feelings of euphoria and competence (the name *heroin* is derived from the user feeling like a hero). These mood-altering properties may, whether consciously or unconsciously, increase the appeal of opioids for self-medicating impaired reward system functioning.^57,58^ A minority of children with depression and possibly fewer young adults receive adequate services from qualified mental health specialists.^59,60,61,62^ Even when treated, depression may be undertreated in young people. After the US Food and Drug Administration’s 2004 black box warning, antidepressant prescriptions for adolescents and young adults declined steeply,^63^ and even when they are prescribed, antidepressants do not necessarily improve rewards-related functioning.^64^

Children with chronic depression may also later take opioids to alleviate the physical symptoms and pain that often accompany depression.^65,66,67^ Depression as a cause of such symptoms may not be evident and thus these complaints may lead to unnecessary opioid prescriptions and first exposure to opioid-associated euphoria.^12,57^ Consistent with work showing that pain and physical health problems often precede long-term opioid use in adults, we found that childhood somatic complaints (and, at the statistical trend level, elevated inflammation and injury) were associated with progression from any to weekly nonheroin opioid use.^68,69,70,71^

### Childhood Substance Use and Later Opioid Use

Consistent with studies that began in later adolescence or adulthood,^18,49,50,72^ our study revealed strong associations between earlier tobacco use and later opioid use. Several mechanisms could be at play. First, adolescent nicotine exposure alters neurodevelopment, changing the developing brain’s reward circuitry and motivational systems.^73,74,75^ This increases opioid-associated reinforcement and stimulation^76,77^ and alters opioid metabolism and efficacy, increasing misuse liability.^78^ Second, adolescent nicotine use or dependence comes with social or health challenges,^76,79,80^ including risk of later depression.^81,82,83^ Third, nicotine use lowers adolescents’ pain thresholds^84^ and increases the risk of health problems for which opioids are often prescribed.^85^ Fourth, adolescent tobacco use and cannabis use are gateways to harder drugs.^18,72^ Adolescents who smoke typically select friends with similar habits, who may provide access to harder drugs.^86^ Finally, unobserved genetic factors could underlie nicotine and cannabis use, depression and rewards system impairments, and opioid use.^87^

### Race/Ethnicity and Opioid Use

American Indian participants showed particularly high rates of weekly nonheroin opioid use, which could be because of early initiation of drug use (eg, cannabis).^88^ Furthermore, in the region of study, American Indian individuals may have better health care access than White individuals because of the Indian Health Services. Easier access combined with greater need for health care (eg, because of poor cardiometabolic health^40,89^) may result in increased contact with health care professionals, who may prescribe opioids.^90^ Finally, American Indian individuals older than 18 years in this sample received cash transfers of approximately $6000 per year, potentially increasing disposable income for drug purchases.^91^

### No Unique Association Between Several Childhood Risk Factors and Later Opioid Use

Several associations were notably absent. Alcohol use by age 16 years was not uniquely associated with opioid use after adjusting for childhood tobacco use and cannabis use. This is consistent with some^50^ but not other^49^ previous work. It is possible that only problematic or very early alcohol use signal risk of later opioid use.^14^ In addition, no or few associations emerged between opioid use and childhood sociodemographic status, maltreatment, family dysfunction, or anxiety. Previous studies typically measured these risk factors retrospectively^92^ or in late adolescence and young adulthood^22,93^ and most did not consider depressive disorders, which may mediate associations between select childhood risk factors and later opioid use.

### Strengths and Limitations

The current study’s prospective longitudinal community-representative psychiatric-diagnostic design has many strengths. For example, prospective assessments from age 9 years, including assessments of childhood adversities or rare instances of substance use, address the problem of retrospective forgetting.^24^ Furthermore, this study is unique in including up to 11 repeated opioid use assessments combined with up to 7 assessments of childhood psychiatric status and adversities.

This study had limitations. First, we were unable to distinguish between medical and nonmedical opioid use. Because nonheroin opioid use was assessed alongside illegal drugs, we likely primarily assessed nonmedical use. Medical and nonmedical use are associated, with many young people initiating nonmedical use following prescribed opioid use.^13^ Second, the example opioids listed in the CAPA/YAPA do not exhaustively reflect those on the market. Additionally, Black individuals were excluded because of low sample size. Notably, their lifetime prevalence of opioid use was low, which is consistent with previous work and likely because of limited access to health care and racial bias in prescribing patterns.^13,94^

## Conclusions

Opioid-related premature mortality of young adults has skyrocketed.^11^ Although prescription practices have changed, no effective solution for the current epidemic or promising preventive measures against future opioid crises are in sight. Our study identified tobacco use and childhood depression by age 16 years as key risk factors of young adult opioid use. Each of these is associated with impaired rewards function, which increases vulnerability to opioid-associated euphoria. Our findings suggest strong opportunities for early prevention and intervention, including in primary care settings.^95,96,97^ Known evidence-based prevention strategies could save lives, especially because mental health and substance use disorders are associated with opioid overdoses among the young.^98^

## Notes

Supplement. **eFigure 1**. Ascertainment of the original Great Smoky Mountains Study sample. **eFigure 2**. Percentage (and standard deviation) of cumulative lifetime any nonheroin opioid use by age in groups defined by sex and race/ethnicity. **eFigure 3**. Percentage (and standard deviation) of cumulative lifetime weekly nonheroin opioid use by age in groups defined by sex and race/ethnicity. **eFigure 4**. Percentage (and standard deviation) of cumulative lifetime heroin use by age in groups defined by sex and race/ethnicity. **eTable 1**. Percentages of childhood risk markers (aggregated across ages 9–16) in groups defined by presence of opioid use by age 30. Cases in the different opioid use groups may overlap. **eTable 2**. Associations between childhood risk markers (between ages 9 and16) and adult lifetime use of opioids (by age 30). For domains 1–6 (starting with sociodemographic variables), associations are adjusted for sex, race, and cohort, but no other variables. N = 1,252. Weighted percentages and unweighted N. **eTable 3**. Interactions with sex. Results from models that tested product terms between each childhood risk factor and child sex. Models were adjusted for race/ethnicity and cohort. P-values tested the significance of the interaction. For significant interactions, models were run separately by sex. **eTable 4**. Interactions with race/ethnicity. Results from models that tested a product term between each childhood risk factor and child race/ethnicity. Models were adjusted for sex and cohort. P-values test the significance of the interaction. For significant interactions, models were run separately for White and American Indian participants. **eTable 5**. Results from multivariate models that entered risk markers within each risk domain simultaneously, adjusting for sex, race/ethnicity, and cohort, and excluding participants who had consumed opioids by age 16. N = 1,229. **eTable 6**. Associations between specific childhood depressive symptoms and opioid use. Each association displayed here is adjusted for sex, race/ethnicity, and cohort. **eTable 7**. Correlates of putative progression to weekly nonheroin opioid use and to heroin use by age 30. Each association displayed here is adjusted for sex, race/ethnicity, and cohort. **eTable 8**. Correlates of putative progression to weekly nonheroin opioid use and heroin use. Results are from multivariate models in which risk markers within each domain were entered simultaneously, adjusting for sex, race/ethnicity, and cohort.
