# Supplementary material for: Prevalence and Childhood Precursors of Opioid Use in the Early Decades of Life
Source: JAMA Pediatr. 2020 Dec 28;175(3):1–10. doi: 10.1001/jamapediatrics.2020.5205 (PMC7770613; doi:10.1001/jamapediatrics.2020.5205)
Supplement: Supplement. — eFigure 1. Ascertainment of the original Great Smoky Mountains Study sample. eFigure 2. Percentage (and standard deviation) of cumulative lifetime any nonheroin opioid use by age in groups defined by sex and race/ethnicity. eFigure 3. Percentage (and standard deviation) of cumulative lifetime weekly nonheroin opioid use by age in groups defined by sex and race/ethnicity. eFigure 4. Percentage (and standard deviation) of cumulative lifetime heroin use by age in groups defined by sex and race/ethnicity. eTable 1. Percentages of childhood risk markers (aggregated across ages 9–16) in groups defined by presence of opioid use by age 30. Cases in the different opioid use groups may overlap. eTable 2. Associations between childhood risk markers (between ages 9 and16) and adult lifetime use of opioids (by age 30). For domains 1–6 (starting with sociodemographic variables), associations are adjusted for sex, race, and cohort, but no other variables. N = 1,252. Weighted percentages and unweighted N. eTable 3. Interactions with sex. Results from models that tested product terms between each childhood risk factor and child sex. Models were adjusted for race/ethnicity and cohort. P-values tested the significance of the interaction. For significant interactions, models were run separately by sex. eTable 4. Interactions with race/ethnicity. Results from models that tested a product term between each childhood risk factor and child race/ethnicity. Models were adjusted for sex and cohort. P-values test the significance of the interaction. For significant interactions, models were run separately for White and American Indian participants. eTable 5. Results from multivariate models that entered risk markers within each risk domain simultaneously, adjusting for sex, race/ethnicity, and cohort, and excluding participants who had consumed opioids by age 16. N = 1,229. eTable 6. Associations between specific childhood depressive symptoms and opioid use. Each association displayed here is [file jamapediatr-e205205-s001.pdf]

# Supplementary Online Content

Shanahan L, Hill SN, Bechtiger L, et al. Prevalence and childhood precursors of opioid use in the early decades of life. *JAMA Pediatr*. Published online December 28, 2020. doi:10.1001/jamapediatrics.2020.5205

**eFigure 1.** Ascertainment of the original Great Smoky Mountains Study sample.

**eFigure 2.** Percentage (and standard deviation) of cumulative lifetime any nonheroin opioid use by age in groups defined by sex and race/ethnicity.

**eFigure 3.** Percentage (and standard deviation) of cumulative lifetime weekly nonheroin opioid use by age in groups defined by sex and race/ethnicity.

**eFigure 4.** Percentage (and standard deviation) of cumulative lifetime heroin use by age in groups defined by sex and race/ethnicity.

**eTable 1.** Percentages of childhood risk markers (aggregated across ages 9–16) in groups defined by presence of opioid use by age 30. Cases in the different opioid use groups may overlap.

**eTable 2.** Associations between childhood risk markers (between ages 9 and16) and adult lifetime use of opioids (by age 30). For domains 1–6 (starting with sociodemographic variables), associations are adjusted for sex, race, and cohort, but no other variables. N = 1,252. Weighted percentages and unweighted N.

**eTable 3.** Interactions with sex. Results from models that tested product terms between each childhood risk factor and child sex. Models were adjusted for race/ethnicity and cohort. P-values tested the significance of the interaction. For significant interactions, models were run separately by sex.

**eTable 4.** Interactions with race/ethnicity. Results from models that tested a product term between each childhood risk factor and child race/ethnicity. Models were adjusted for sex and cohort. P-values test the significance of the interaction. For significant interactions, models were run separately for White and American Indian participants.

**eTable 5.** Results from multivariate models that entered risk markers within each risk domain simultaneously, adjusting for sex, race/ethnicity, and cohort, and excluding participants who had consumed opioids by age 16. N = 1,229.

© 2020 Shanahan L et al. *JAMA Pediatrics.*

**eTable 6.** Associations between specific childhood depressive symptoms and opioid use. Each association displayed here is adjusted for sex, race/ethnicity, and cohort.

**eTable 7.** Correlates of putative progression to weekly nonheroin opioid use and to heroin use by age 30. Each association displayed here is adjusted for sex, race/ethnicity, and cohort.

**eTable 8.** Correlates of putative progression to weekly nonheroin opioid use and heroin use. Results are from multivariate models in which risk markers within each domain were entered simultaneously, adjusting for sex, race/ethnicity, and cohort.

This supplementary material has been provided by the authors to give readers additional information about their work.

© 2020 Shanahan L et al. *JAMA Pediatrics.*

## Ascertainment of Great Smoky Mountains Study Sample

**eFigure 1.** Ascertainment of the original Great Smoky Mountains Study sample

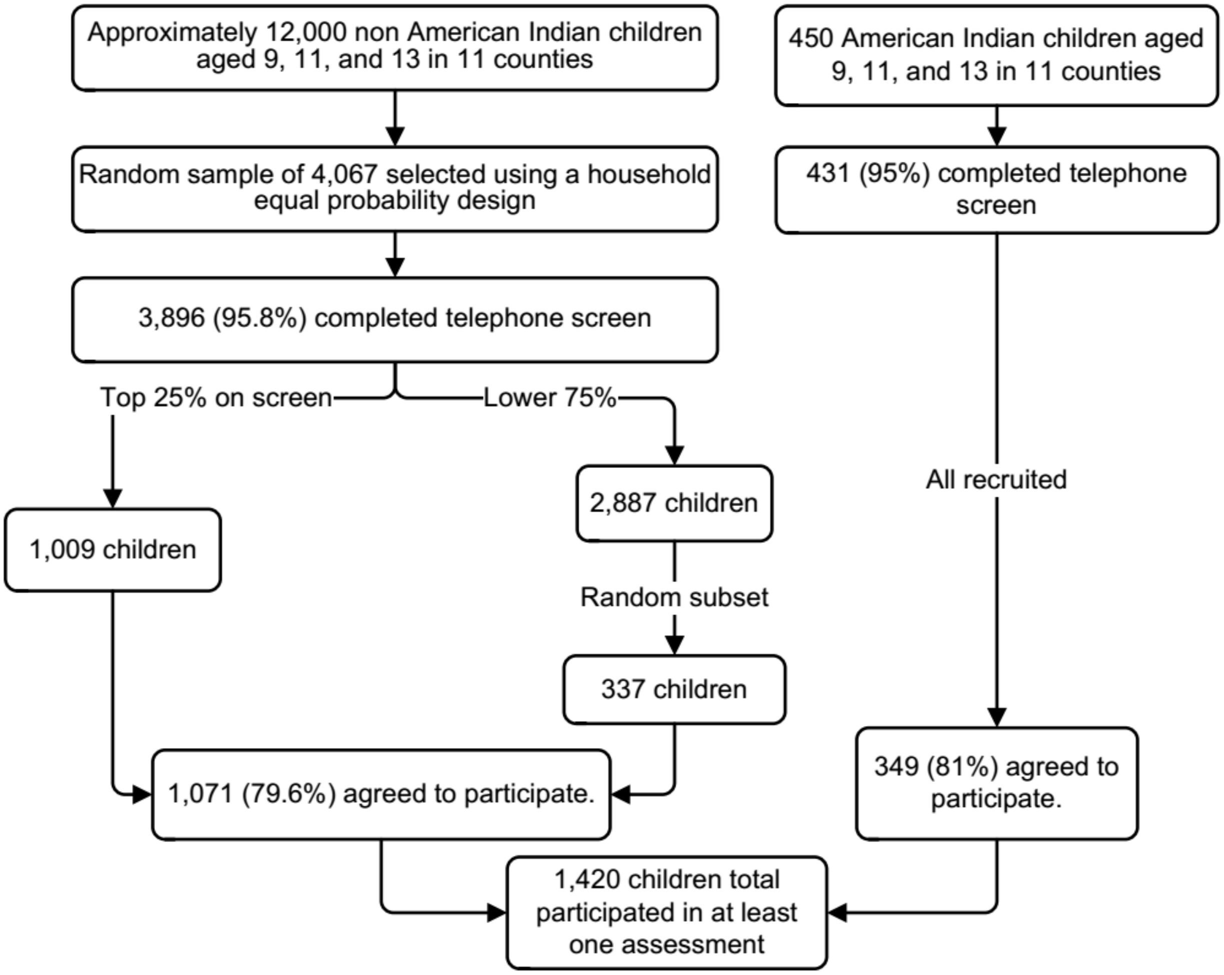


© 2020 Shanahan L et al. *JAMA Pediatrics.*

## Prevalence of Opioid Use by Race/Ethnicity and Sex

**eFigure 2.** Percentage (and standard deviation) of cumulative lifetime any nonheroin opioid use by age in groups defined by sex and race/ethnicity.

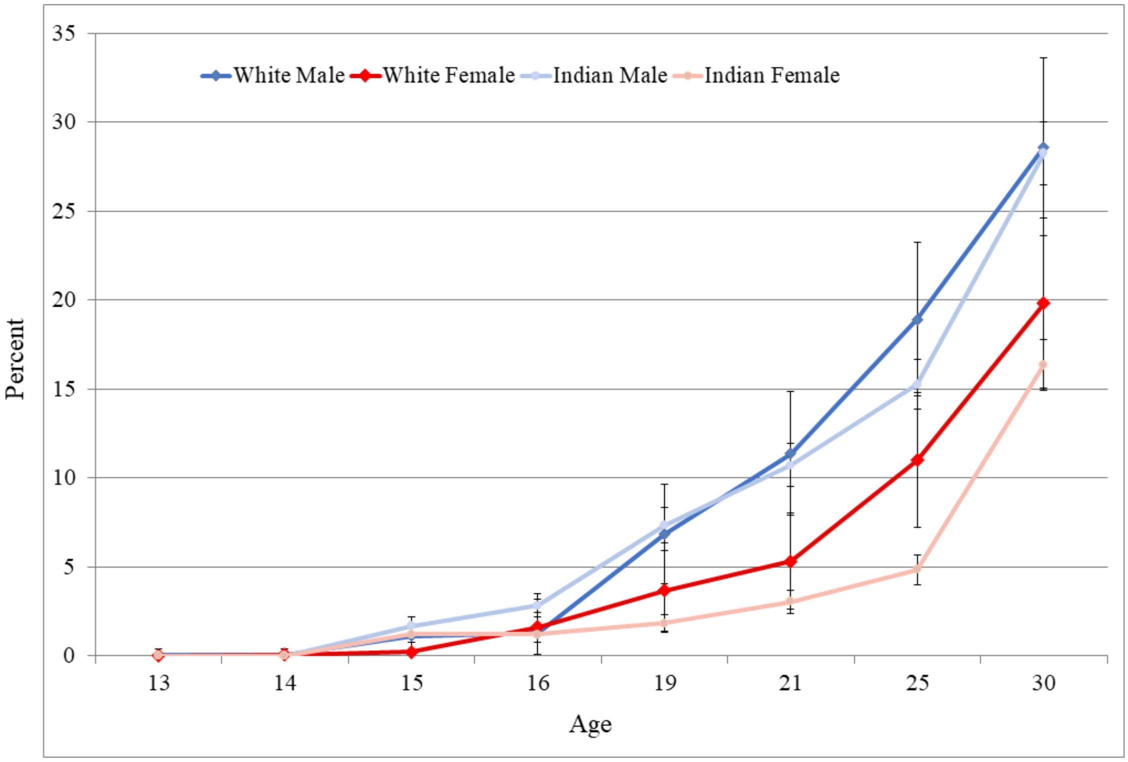


**eFigure 2** shows the cumulative lifetime prevalence of non-heroin opioid use within groups defined by race/ethnicity and sex. By age 30, cumulative lifetime non-heroin opioid use was higher in males than in females (OR = 1.63, CI: 1.05–2.53, p = .029). This sex difference became significant by age 21.

**eFigure 3** shows that by age 30, cumulative lifetime weekly non-heroin opioid use was higher in American Indian than in White participants (OR = 1.89, 95% CI: 1.22–2.93, p = .005). American Indian males had the highest rates of weekly or daily non-heroin opioid use beginning at an early age, and by age 30, almost one in five American Indian males had taken non-heroin opioids weekly or daily. Both American Indian males and females displayed particularly steep increases in cumulative lifetime rates of weekly use from ages 25 to 30.

© 2020 Shanahan L et al. *JAMA Pediatrics.*

**eFigure 3.** Percentage (and standard deviation) of cumulative lifetime weekly nonheroin opioid use by age in groups defined by sex and race/ethnicity.

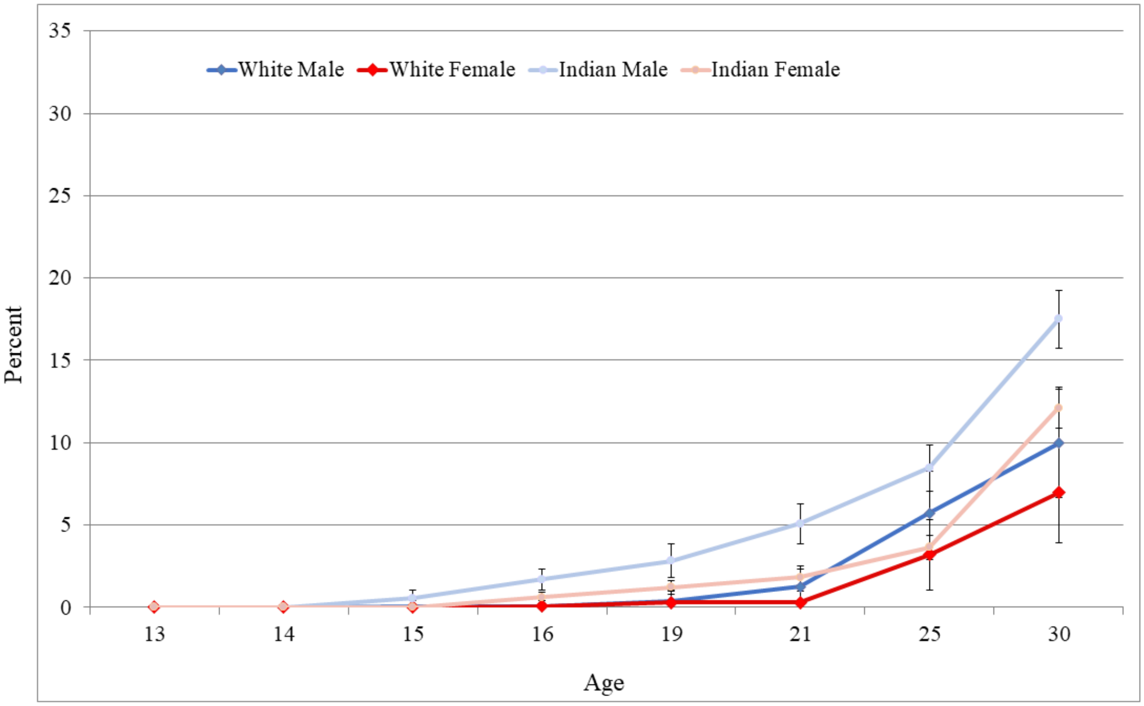


**eFigure 4** shows that cumulative lifetime heroin use at age 30 was higher in males than in females (OR = 2.29, CI: 1.02–5.17, p = .045) and particularly high in American Indian males. Indeed, analyses *within* the American Indian group showed that males had a higher cumulative lifetime prevalence rate of heroin use by age 30 than females (OR = 2.64, CI: 1.14–6.14, p = .024). A sex by race/ethnicity interaction term was not significant, however.

© 2020 Shanahan L et al. *JAMA Pediatrics.*

**eFigure 4.** Percentage (and standard deviation) of cumulative lifetime heroin use by age in groups defined by sex and race/ethnicity.

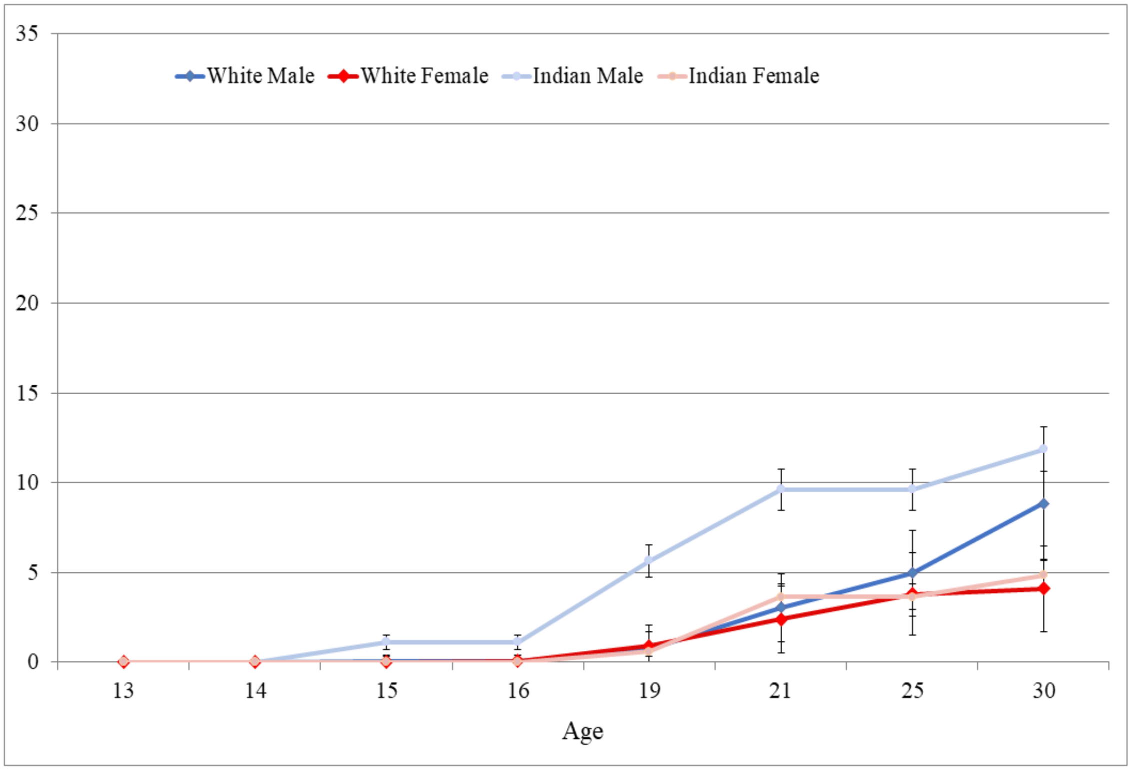


Taken together, results revealed that males and females differ in their lifetime use of non-heroin opioid and heroin use by age 30. Significant race/ethnicity differences emerged for weekly non-heroin opioid use only. American Indian males had the highest lifetime prevalence of weekly non-heroin opioid and heroin use, with sex differences in cumulative lifetime heroin use becoming significant within the American Indian group by age 30.

© 2020 Shanahan L et al. *JAMA Pediatrics.*

## Prevalence of Risk in No Opioid Use and Opioid Use Groups

**eTable 1.** Percentages of childhood risk markers (aggregated across ages 9–16) in groups defined by presence of opioid use by age 30. Cases in the different opioid use groups may overlap.

| | **No Lifetime Opioid Use** N = 911 | **Any Non-Heroin Opioid Use** N = 322 | **Weekly Non-Heroin Opioid Use** N = 155 | **Any Heroin Use** N = 95 |
|---|---|---|---|---|
| **0. Cohort, sex, race/ethnicity** | **%** | **%** | **%** | **%** |
| Male | 47.0 | 59.5 | 59.2 | 68.8 |
| American Indian | 4.2 | 3.8 | 7.0 | 5.3 |
| Cohort 1 | 33.5 | 44.0 | 47.4 | 35.4 |
| Cohort 2 | 38.5 | 24.3 | 29.3 | 41.6 |
| **1. Sociodemographics/family** | | | | |
| Family low SES | 26.1 | 28.2 | 33.1 | 25.3 |
| Family instability | 22.6 | 29.6 | 25.4 | 40.2 |
| Family dysfunction | 12.3 | 17.8 | 19.8 | 14.0 |
| Maltreatment | 28.6 | 35.2 | 39.4 | 44.6 |
| **2. Child's school and peer risk** | | | | |
| Expelled from school | 3.2 | 8.3 | 10.7 | 12.2 |
| Peers exhibiting social deviance | 28.7 | 48.1 | 59.7 | 43.6 |
| Mostly older friends (2+ yrs) | 9.5 | 19.9 | 15.1 | 16.3 |
| Victim of bullying | 27.0 | 38.4 | 42.9 | 44.1 |
| **3. Parental mental illness, drug problems, legal involvement** | | | | |
| Parental mental health service use | 45.0 | 58.3 | 47.1 | 61.3 |
| Parental drug-related service use | 14.7 | 18.4 | 25.4 | 24.5 |
| Parental legal involvement | 38.2 | 52.8 | 65.0 | 44.7 |
| **4. Child's substance use** | | | | |
| Smoking/tobacco use | 13.5 | 48.7 | 65.7 | 63.5 |
| Alcohol use | 14.4 | 36.8 | 41.8 | 49.0 |
| Cannabis use | 6.6 | 31.2 | 37.2 | 44.5 |
| Other illicit drug use | 1.0 | 6.2 | 4.8 | 15.8 |
| **5. Child's psychiatric risk** | | | | |
| Anxiety disorders | 8.6 | 15.1 | 17.6 | 26.3 |
| Depressive disorders | 5.3 | 15.3 | 26.2 | 35.3 |
| Oppositional defiant disorder | 11.6 | 17.9 | 31.3 | 30.0 |
| Conduct disorder | 5.4 | 15.1 | 21.6 | 29.6 |
| ADHD | 3.0 | 5.0 | 7.1 | 8.8 |
| Comorbidity: ≥2 diagnoses | 8.5 | 23.8 | 34.0 | 45.7 |
| **6. Child's physical health risks** | | | | |
| Obesity | 28.0 | 30.3 | 30.8 | 34.5 |
| Somatic complaints | 30.5 | 30.6 | 42.8 | 50.8 |
| Injury | 40.0 | 47.1 | 57.3 | 57.5 |
| Inflammation: CRP ≥3 mg/L | 18.4 | 29.7 | 42.8 | 36.5 |

© 2020 Shanahan L et al. *JAMA Pediatrics.*

## Associations Between Childhood Risk and Lifetime Opioid Use

**eTable 2.** Associations between childhood risk markers (between ages 9 and16) and adult lifetime use of opioids (by age 30). For domains 1–6 (starting with sociodemographic variables), associations are adjusted for sex, race, and cohort, but no other variables. N = 1,252. Weighted percentages and unweighted N.

| **Childhood Risk Factors** | **Prevalence of Risk** | | **Any Non-Heroin Opioid Use** N = 322, 24.2% | | **Weekly Non-Heroin Opioid Use** N = 155, 8.8% | | **Any Heroin Use** N = 95, 6.6% | |
|---|---|---|---|---|---|---|---|---|
| **0. Sex, race/ethnicity, cohort** | **%** | **N** | **OR** | **95% CI** | **OR** | **95% CI** | **OR** | **95% CI** |
| Sex (1 = male) | 50.3 | 677 | **1.63*** | 1.05–2.53 | 1.48 | 0.80–2.74 | **2.29*** | 1.02–5.17 |
| American Indian (1 = AI) | 4.1 | 342 | 0.91 | 0.65–1.27 | **1.89**** | 1.22–2.93 | 1.34 | 0.78–2.31 |
| Cohort 1 (ref = cohort 3) | 35.7 | 437 | 1.18 | 0.71–1.98 | 1.73 | 0.81–3.73 | 1.27 | 0.49–3.29 |
| Cohort 2 (ref = cohort 3) | 35.4 | 448 | **0.55*** | 0.31–0.97 | 1.03 | 0.45–2.33 | 1.53 | 0.60–3.88 |
| **1. Sociodemographics & family** | | | | | | | | |
| Family low socioeconomic status | 26.7 | 491 | 1.04 | 0.65–1.67 | 1.27 | 0.65–2.47 | 0.88 | 0.37–2.08 |
| Family instability | 24.2 | 377 | **1.54#** | 0.95–2.50 | 1.03 | 0.55–1.90 | **2.32*** | 1.08–4.95 |
| Family dysfunction | 13.6 | 214 | 1.56 | 0.88–2.77 | 1.65 | 0.83–3.29 | 1.08 | 0.45–2.61 |
| Maltreatment | 31.0 | 503 | **1.46#** | 0.94–2.28 | 1.60 | 0.89–2.90 | 1.85 | 0.85–4.01 |
| **2. Child's school and peer risk** | | | | | | | | |
| Expelled from school | 4.5 | 86 | **2.51*** | 1.12–5.66 | **2.81*** | 1.15–6.88 | **3.15*** | 1.20–8.24 |
| Peers exhibiting social deviance | 33.5 | 528 | **2.66***** | 1.66–4.25 | **4.27***** | 2.27–8.01 | 1.75 | 0.83–3.71 |
| Mostly older friends (2+ yrs) | 12.3 | 220 | **2.20**** | 1.25–3.86 | 1.15 | 0.54–2.44 | 1.45 | 0.60–3.53 |
| Victim of bullying | 30.2 | 432 | **1.63*** | 1.03–2.59 | **1.73#** | 0.95–3.17 | **1.88#** | 0.90–3.94 |
| **3. Parental mental illness, drug problems, legal involvement** | | | | | | | | |
| Par. mental health service use | 48.5 | 642 | **1.79*** | 1.15–2.81 | 1.10 | 0.58–2.07 | **2.02#** | 0.91–4.49 |
| Parental drug service use | 15.8 | 299 | 1.34 | 0.80–2.24 | **1.87#** | 0.98–3.56 | **2.31*** | 1.05–5.11 |
| Parental legal involvement | 41.5 | 661 | **2.04**** | 1.31–3.17 | **2.72**** | 1.38–5.39 | 1.04 | 0.50–2.19 |

© 2020 Shanahan L et al. *JAMA Pediatrics.*

| | | | | | | | | |
|---|---|---|---|---|---|---|---|---|
| **4. Child's substance use** | | | | | | | | |
| Smoking/tobacco use | 22.3 | 364 | **5.98*****| 3.69–9.71 | **8.59***** | 4.54–16.26 | **6.92***** | 3.26–14.70 |
| Alcohol use | 20.2 | 285 | **3.73***** | 2.26–6.17 | **3.48***** | 1.83–6.63 | **4.49***** | 2.10–9.57 |
| Cannabis use | 12.9 | 208 | **6.58***** | 3.60–12.05 | **5.09***** | 2.59–10.00 | **6.56***** | 2.99–14.38 |
| Other illicit drug use | 2.6 | 51 | **5.83**** | 1.93–17.65 | **2.33*** | 1.06–5.12 | **12.07***** | 4.03–36.15 |
| **5. Child's psychiatric risk** | | | | | | | | |
| Anxiety disorders | 10.8 | 176 | **1.76#** | 0.96–3.24 | 1.87 | 0.82–4.24 | **3.61**** | 1.47–8.86 |
| Depressive disorders | 8.4 | 128 | **3.15***** | 1.61–6.18 | **5.43***** | 2.49–11.84 | **9.03***** | 3.77–21.63 |
| Oppositional defiant disorder | 13.5 | 270 | **1.60#** | 0.97–2.66 | **3.40***** | 1.70–6.81 | **2.71**** | 1.27–5.78 |
| Conduct disorder | 8.1 | 166 | **2.85***** | 1.59–5.12 | **3.86***** | 1.86–8.02 | **5.30***** | 2.51–11.21 |
| ADHD | 3.5 | 69 | 1.28 | 0.50–3.30 | 1.78 | 0.53–5.93 | 2.47 | 0.67–9.14 |
| Comorbidity: ≥2 diagnoses | 12.9 | 247 | **2.98***** | 1.76–5.07 | **4.05***** | 2.10–7.82 | **6.87***** | 3.25–14.52 |
| **6. Child's physical health** | | | | | | | | |
| Obesity | 28.6 | 464 | 1.02 | 0.63–1.66 | 0.96 | 0.77–3.72 | 1.21 | 0.59–2.50 |
| Somatic complaints | 31.2 | 410 | 1.06 | 0.67–1.69 | **1.93*** | 1.03–3.60 | **2.80*** | 1.23–6.34 |
| Injury | 42.4 | 541 | 1.27 | 0.82–1.96 | **1.83#** | 0.97–3.45 | 1.82 | 0.83–3.99 |
| Inflammation at CRP ≥ 3 mg/L | 21.2 | 312 | **1.83*** | 1.09–3.07 | **2.97**** | 1.50–5.88 | **2.19#** | 0.98–4.90 |

Significance: #p < .10 *p < .05 **p < .01 ***p < .001

© 2020 Shanahan L et al. *JAMA Pediatrics.*

## Testing Interactions of Sex and Race/Ethnicity with Childhood Risk Factors in the Prediction of Young Adult Opioid Use

**eTable 3.** Interactions with sex. Results from models that tested product terms between each childhood risk factor and child sex. Models were adjusted for race/ethnicity and cohort. P-values tested the significance of the interaction. For significant interactions, models were run separately by sex.

| **Childhood Risk Factors** | **Any Non-Heroin Opioid Use** N = 322, 24.2% | | **Weekly Non-Heroin Opioid Use** N = 155, 8.8% | | **Any Heroin Use** N = 95, 6.6% | |
|---|---|---|---|---|---|---|
| **1. Sociodemographics/family** | *p* | Odds Ratio | *p* | Odds Ratio | *p* | Odds Ratio |
| Family low SES | .506 | | .648 | | .648 | |
| Family instability | .154 | | .360 | | .250 | |
| Family dysfunction | **.037** | **F: 2.75*** M: 0.84 | **.010** | **F: 3.65*** M: 0.69 | **.013** | **F: 0.20*** M: 1.68 |
| Maltreatment | .146 | | .083 | | .661 | |
| **2. School/peer risk** | | | | | | |
| Expelled from school | .938 | | .773 | | .683 | |
| Peers exhibiting social deviance | .727 | | .339 | | .770 | |
| Mostly older friends (2+ yrs) | .140 | | .117 | | .475 | |
| Victim of bullying | .483 | | .295 | | .103 | |
| **3. Parental mental illness, drug problems, legal involvement** | | | | | | |
| Par. mental health service use | **.046** | **F: 3.00**** M: 1.19 | **.018** | **F: 2.78#** M: 0.59 | .409 | |
| Parental drug service use | .126 | | .432 | | .093 | |
| Parental legal involvement | .158 | | **.036** | **F: 6.57***** M: 1.65 | .205 | |
| **4. Child's substance use** | | | | | | |
| Smoking/tobacco use | .200 | | **.020** | **F: 8.15*** M: 4.71***** | **.013** | F: 1.88 **M: 15.24***** |
| Alcohol use | .250 | | .216 | | .741 | |
| Cannabis use | .236 | | .202 | | .858 | |
| Other illicit drug use | .348 | | .335 | | .887 | |
| **5. Child's psychiatric risk** | | | | | | |
| Anxiety disorders | .980 | | .867 | | **.009** | F: 0.90 **M: 6.59***** |
| Depressive disorders | .846 | | .363 | | .313 | |
| Oppositional defiant disorder | .119 | | **.006** | F: 1.88 **M: 15.24***** | .139 | |
| Conduct disorder | .544 | | .428 | | .135 | |
| ADHD | .538 | | .633 | | .633 | |
| Comorbidity: ≥2 diagnoses | .328 | | .515 | | .271 | |
| **6. Child's physical health risks** | | | | | | |
| Obesity | .622 | | .496 | | .063 | |
| Somatic complaints | .956 | | .902 | | **.044** | F: 0.79 **M: 5.23***** |
| Injury | .582 | | .938 | | .810 | |
| Inflammation: CRP ≥3 mg/L | .767 | | .831 | | .589 | |

Significance: #p < .10 *p < .05 **p < .01 ***p < .001

© 2020 Shanahan L et al. *JAMA Pediatrics.*

**eTable 4.** Interactions with race/ethnicity. Results from models that tested a product term between each childhood risk factor and child race/ethnicity. Models were adjusted for sex and cohort. P-values test the significance of the interaction. For significant interactions, models were run separately for White and American Indian participants.

| **Childhood Risk Factors** | **Any Non-Heroin Opioid Use**<br>N = 322, 24.2% | | **Weekly Non-Heroin Opioid Use**<br>N = 155, 8.8% | | **Any Heroin Use**<br>N = 95, 6.6% | |
|---|---|---|---|---|---|---|
| **1. Sociodemographics/family** | *p* | Odds Ratio | *p* | Odds Ratio | *p* | Odds Ratio |
| Family low-SES | .863 | | .338 | | .977 | |
| Family instability | .188 | | .883 | | .108 | |
| Family dysfunction | .884 | | .461 | | **.011** | W: 0.95<br>**AI: 5.14*** ** |
| Maltreatment | .867 | | .439 | | .678 | |
| **2. School/peer risk** | | | | | | |
| Expelled from school | .736 | | .627 | | .427 | |
| Peers exhibiting social deviance | .345 | | .403 | | .791 | |
| Mostly older friends (2+ yrs) | .755 | | .123 | | .954 | |
| Victim of bullying | **.041** | **W: 1.67***<br>AI: 0.71 | **.022** | **W: 1.83#**<br>AI: 0.53 | **.027** | W: **1.99#**<br>AI: 0.36 |
| **3. Parental mental illness, drug problems, legal involvement** | | | | | | |
| Par. mental health service use | .892 | | .140 | | .735 | |
| Parental drug service use | .656 | | .866 | | .605 | |
| Parental legal involvement | .600 | | .194 | | .639 | |
| **4. Child's substance use** | | | | | | |
| Smoking/tobacco use | **.027** | **W: 6.23*****<br>**AI: 2.49**** | .103 | | .056 | |
| Alcohol use | .709 | | .958 | | .417 | |
| Cannabis use | .451 | | .999 | | .673 | |
| Other illicit drug use | .390 | | **.005** | W: 1.77<br>**AI: 14.82***** | .052 | |
| **5. Child's psychiatric risk** | | | | | | |
| Anxiety disorders | .809 | | .835 | | .234 | |
| Depressive disorders | .220 | | .070 | | **.024** | **W: 9.64*****<br>AI: 1.62 |
| Oppositional defiant disorder | .533 | | **.041** | **W: 3.62*****<br>AI: 1.17 | .954 | |
| Conduct disorder | .132 | | .361 | | .644 | |
| ADHD | .372 | | .424 | | -- | |
| Comorbidity: ≥2 diagnoses | .375 | | .449 | | .103 | |
| **6. Child's physical health** | | | | | | |
| Obesity | .979 | | .662 | | .952 | |
| Somatic complaints | .463 | | .291 | | .686 | |
| Injury | .354 | | .822 | | .231 | |
| Inflammation: CRP ≥3 mg/L | **.028** | **W: 1.91*****<br>AI: 0.85 | **.011** | **W: 3.23*****<br>AI: 0.99 | .513 | |

Significance: #p < .10 *p < .05 **p < .01 ***p < .001

© 2020 Shanahan L et al. *JAMA Pediatrics.*

## Excluding Cases with Any Opioid/Heroin Use by Age 16

**eTable 5.** Results from multivariate models that entered risk markers within each risk domain simultaneously, adjusting for sex, race/ethnicity, and cohort, and excluding participants who had consumed opioids by age 16. N = 1,229.

| Childhood Risk Factors | Any Non-Heroin Opioid Use N = 299, 23.0% | | Weekly Non-Heroin Opioid Use N = 140, 8.2% | | Any Heroin Use N = 86, 5.5% | |
|---|---|---|---|---|---|---|
| **1. Sociodemographics/family** | **OR** | **95% CI** | **OR** | **95% CI** | **OR** | **95% CI** |
| Family low SES | 0.88 | 0.52–1.49 | 0.92 | 0.42–2.00 | 0.59 | 0.24–1.44 |
| Family instability | 1.39 | 0.83–2.34 | 0.86 | 0.43–1.72 | **2.15** | 0.84–5.50 |
| Family dysfunction | 1.40 | 0.74–2.64 | 1.30 | 0.58–2.29 | 0.89 | 0.33–2.40 |
| Maltreatment | 1.25 | 0.74–2.09 | 1.66 | 0.81–3.40 | **2.17** | 0.87–5.29 |
| **2. Child's school/peer risk** | | | | | | |
| Expelled from school | 1.25 | 0.50–3.11 | 1.40 | 0.45–4.37 | **2.66#** | 0.84–8.35 |
| Peers exhibit social deviance | **2.49***** | 1.52–4.09 | **3.59***** | 1.84–6.99 | 1.51 | 0.67–3.40 |
| Mostly older friends (2+ years) | **1.95*** | 1.08–3.53 | 0.93 | 0.41–2.08 | 1.27 | 0.46–3.49 |
| Victim of bullying | 1.51 | 0.94–2.44 | 1.46 | 0.76–2.79 | 1.99 | 0.87–4.56 |
| **3. Parental MI, drug, legal inv.** | | | | | | |
| Par. mental health service use | **1.69*** | 1.04–2.74 | 0.82 | 0.41–1.64 | **2.59*** | 1.00–6.66 |
| Parental drug service use | 0.77 | 0.44–1.37 | 1.27 | 0.61–2.64 | **2.37#** | 0.86–6.50 |
| Parental legal involvement | **1.87**** | 1.14–3.07 | **3.02**** | 1.47–6.22 | 0.52 | 0.18–1.47 |
| **4. Child's substance use** | | | | | | |
| Smoking/tobacco use | **4.03***** | 2.28–7.12 | **6.73***** | 3.22–14.05 | **4.74***** | 1.97–11.42 |
| Alcohol use | 1.24 | 0.60–2.57 | 1.06 | 0.42–2.67 | 1.09 | 0.41–2.89 |
| Cannabis use | **2.40**** | 1.11–5.16 | 1.79 | 0.64–5.01 | **1.49** | 0.51–4.39 |
| Other illicit drug use | 0.80 | 0.25–2.52 | 0.50 | 0.18–1.39 | **2.92#** | 0.96–8.89 |
| **5. Child's psychiatric risk** | | | | | | |
| Anxiety disorders | 1.16 | 0.57–2.33 | 0.65 | 0.21–1.99 | 1.60 | 0.56–4.63 |
| Depressive disorders | **2.05#** | 0.96–4.37 | **3.75*** | 1.30–10.80 | **4.75*** | 1.39–16.20 |
| Oppositional defiant disorder | 1.06 | 0.55–2.06 | **2.14** | 0.78–5.89 | 1.31 | 0.54–3.19 |
| Conduct disorder | **2.10*** | 1.08–4.06 | **1.82** | 0.65–5.12 | **2.94*** | 1.13–7.63 |
| ADHD | 0.95 | 0.34–2.65 | 0.84 | 0.29–2.41 | 1.15 | 0.31–4.24 |
| Comorbidity: ≥2 diagnoses[1] | -- | -- | -- | -- | -- | -- |
| **6. Child's physical health** | | | | | | |
| Obesity | 0.88 | 0.53–1.48 | 0.70 | 0.33–1.45 | 1.11 | 0.54–2.28 |
| Somatic complaints | 1.15 | 0.71–1.84 | **1.89*** | 1.01–3.53 | **4.03***** | 1.85–8.77 |
| Injury | 1.07 | 0.67–1.70 | 1.73 | 0.90–3.32 | 1.76 | 0.77–4.02 |
| Inflammation: CRP ≥3 mg/L | **1.66#** | 0.98–2.80 | **2.75**** | 1.34–5.66 | 1.30 | 0.60–2.83 |

Significance: #p < .10 *p < .05 **p < .01 ***p < .001
Odds ratios significant at p < .10 or OR ≥ 2 are bolded. MI=mental illness.
[1] Not included in multivariate models.

© 2020 Shanahan L et al. *JAMA Pediatrics.*

## Associations of Individual Depressive Symptoms with Opioid Use

**eTable 6.** Associations between specific childhood depressive symptoms and opioid use. Each association displayed here is adjusted for sex, race/ethnicity, and cohort.

| | **Prevalence** | | **Any Non-Heroin Opioid Use** N = 322, 24.2% | | **Weekly Non-Heroin Opioid Use** N = 155, 8.8% | | **Any Heroin Use** N = 95, 6.6% | |
|---|---|---|---|---|---|---|---|---|
| | **%** | **N** | **OR** | **95%CI** | **OR** | **95%CI** | **OR** | **95%CI** |
| **Symptoms of Major Depression** | | | | | | | | |
| Depressed/irritable mood | 14.3 | 200 | **2.59*** ** | 1.47–4.45 | **2.82**** | 1.37–5.80 | **6.50*** ** | 2.90–14.56 |
| Anhedonia, loss of interest | 2.2 | 32 | 2.54 | 0.72–8.95 | **7.20**** | 1.99–25.95 | **8.22**** | 2.34–28.79 |
| Weight loss/gain, appetite disturbed[1] | 77.9 | 974 | **2.34**** | 1.27–4.32 | 2.04 | 0.79–5.24 | **5.38*** ** | 2.46–11.77 |
| Insomnia/hypersomnia, nearly daily | 9.2 | 135 | **1.73#** | 0.89–3.36 | **2.35#** | 0.99–5.60 | **2.55#** | 0.94–6.95 |
| Motoric agitation/retardation | 1.4 | 21 | 0.66 | 0.20–2.19 | 1.30 | 0.36–4.73 | **0.12#** | 0.01–1.10 |
| Fatigued/no energy[1] | 14.1 | 174 | 1.17 | 0.62–2.20 | 1.06 | 0.47–2.37 | 1.91 | 0.74–5.00 |
| Feeling worthlessness or guilty | 17.6 | 264 | **2.59*** ** | 1.55–4.32 | **2.81**** | 1.45–5.43 | **5.60*** ** | 2.49–12.60 |
| Problems thinking/making decisions[1] | 4.8 | 85 | **2.00#** | 0.88–4.52 | **3.56**** | 1.49–8.49 | **4.15*** | 1.38–13.02 |
| Suicidal ideation, plan, or attempt | 14.3 | 197 | 1.30 | 0.71–2.36 | **2.26*** | 1.04–4.87 | **2.85*** | 1.16–6.97 |
| **Symptoms of Dysthymia** | | | | | | | | |
| Chronic low mood | 18.0 | 239 | **2.84*** ** | 1.68–4.81 | **3.45*** ** | 1.76–6.74 | **5.12*** ** | 2.26–11.64 |
| Insomnia/hypersomnia | 12.7 | 179 | **1.64#** | 0.92–2.90 | **2.41*** | 1.14–5.11 | **3.68**** | 1.60–8.46 |
| Low self-esteem | 14.5 | 221 | **3.33*** ** | 1.93–5.72 | **3.37*** ** | 1.68–6.74 | **7.31*** ** | 3.16–16.94 |
| Hopelessness | 3.6 | 51 | **2.82*** | 1.10–7.23 | 2.02 | 0.55–7.48 | **4.22*** | 1.20–14.79 |

Significance: #p < .10 *p < .05 **p < .01 ***p < .001

[1] Also a symptom of dysthymia but not listed again in the dysthymia section

© 2020 Shanahan L et al. *JAMA Pediatrics.*

## Correlates of Putative Progression to More Frequent or Illegal Opioid Use

**eTable 7.** Correlates of putative progression to weekly nonheroin opioid use and to heroin use by age 30. Each association displayed here is adjusted for sex, race/ethnicity and cohort.

| Childhood Risk Factors | Any (N = 171) versus Weekly (N = 151) Non-Heroin Opioid Use<br>Weekly = 1 | | Weekly Non-Heroin Opioid (N = 103) vs. Heroin Use (N = 52)<br>Heroin = 1 | |
|---|---|---|---|---|
| **0. Sex, race/ethnicity, cohort** | **OR** | **95% CI** | **OR** | **95% CI** |
| Sex (1 = male) | 1.02 | 0.47–2.24 | **3.65*** | 1.04-12.78 |
| American Indian (1 = AI) | **3.43***** | 1.82–6.43 | 1.09 | 0.43-2.75 |
| Cohort 1 (ref = cohort 3) | 1.82 | 0.72–4.58 | 1.59 | 0.33-7.61 |
| Cohort 2 (ref = cohort 3) | 2.04 | 0.73–5.75 | 1.22 | 0.24-6.21 |
| **1. Sociodemographics/family** | | | | |
| Family low socioeconomic status | 1.28 | 0.53–3.08 | 0.66 | 0.20-2.24 |
| Family instability | 0.68 | 0.31–1.48 | **11.02***** | 3.59-33.79 |
| Family dysfunction | 1.22 | 0.49–3.02 | 0.47 | 0.14-1.59 |
| Maltreatment | 1.18 | 0.56–2.49 | 0.83 | 0.24-2.85 |
| **2. Child's school/peer risk** | | | | |
| Expelled from school | 1.97 | 0.63–6.19 | **5.57*** | 1.33-23.38 |
| Peers exhibiting social deviance | **2.72*** | 1.22–6.06 | 1.94 | 0.47-7.99 |
| Mostly older friends (2+ years) | 0.54 | 0.22–1.32 | 2.40 | 0.79-7.28 |
| Victim of bullying | 1.34 | 0.61–2.93 | **7.01**** | 2.25-21.83 |
| **3. Parental mental illness, drug problems, legal involvement** | | | | |
| Par. mental health service use | 0.53 | 0.23–1.18 | 1.56 | 0.48-5.08 |
| Parental drug service use | 1.92 | 0.80–4.61 | 1.24 | 0.34-4.57 |
| Parent with legal involvement | 1.82 | 0.81–4.09 | 1.46 | 0.36-5.89 |
| **4. Child's substance use** | | | | |
| Smoking/tobacco use | **3.42**** | 1.60–7.29 | 2.59 | 0.51-13.14 |
| Alcohol use | 1.48 | 0.67–3.23 | **5.74*** | 1.21-27.15 |
| Cannabis use | 1.53 | 0.68–3.44 | 2.02 | 0.57-7.13 |
| Other illicit drug use | 0.64 | 0.22–1.83 | 2.04 | 0.58-7.24 |
| **5. Child's psychiatric risk** | | | | |
| Anxiety disorders | 1.34 | 0.49–3.71 | 1.38 | 0.25-7.73 |
| Depressive disorders | **3.28*** | 1.12–9.58 | 1.72 | 0.46-6.39 |
| Oppositional defiant disorder | **3.80**** | 1.64–8.78 | 1.71 | 0.51-5.70 |
| Conduct disorder | **2.49*** | 1.04–5.95 | **8.29**** | 2.29-30.03 |
| ADHD | 1.80 | 0.34–9.50 | **7.59*** | 1.14-50.47 |
| Comorbidity: 2+ diagnoses | **2.25#** | 0.97–5.23 | **3.13#** | 0.90-10.90 |
| **6. Child's physical health risks** | | | | |
| Obesity | 0.89 | 0.37–2.09 | 2.44 | 0.75-7.98 |
| Somatic complaints | **2.56*** | 1.14–5.74 | **4.06*** | 1.37-11.99 |
| Injury | **2.02#** | 0.88–4.67 | 2.52 | 0.83-7.62 |
| Inflammation: CRP ≥3 mg/L | **2.46#** | 0.99–6.15 | 1.25 | 0.32-4.97 |

Significance: #p < .10 *p < .05 **p < .01 ***p < .001

<u>Note:</u> The weekly non-heroin opioid use versus heroin use comparison includes only those heroin users who had used non-heroin opioids weekly. This coding scheme was chosen to better capture progression from less severe to more severe opioid use. Heroin users who had never used non-heroin opioids weekly were not

© 2020 Shanahan L et al. *JAMA Pediatrics.*

included. An underlying assumption of these comparisons is that those with weekly non-heroin opioid use progressed from any non-heroin opioid use and that those with heroin use progressed from weekly non-heroin opioid use. However, it is possible that participants were co-using (i.e., endorsed the two categories of use at the same point in time rather than at sequential points in time) or using in these categories in the "reverse temporal order."

© 2020 Shanahan L et al. *JAMA Pediatrics.*

**eTable 8.** Correlates of putative progression to weekly nonheroin opioid use and heroin. Results are from multivariate models in which risk markers within each domain were entered simultaneously, adjusting for sex, race/ethnicity, and cohort.

| **Childhood Risk Factors** | **Any (N = 151) versus Weekly (N = 171) Non-Heroin Opioid Use** Weekly = 1 | | **Weekly Non-Heroin (N = 103) vs. Heroin use (N = 52)** Heroin = 1 | |
|---|---|---|---|---|
| **1. Sociodemographics/family** | **OR** | **95% CI** | **OR** | **95% CI** |
| Family low socioeconomic status | 1.30 | 0.52–3.24 | 0.48 | 0.11–2.09 |
| Family instability | 0.61 | 0.27–1.36 | **14.06*** ** | 4.27–46.35 |
| Family dysfunction | 1.13 | 0.44–2.90 | 0.57 | 0.18–1.80 |
| Maltreatment | 1.26 | 0.58–2.77 | 0.55 | 0.17–1.75 |
| **2. Child's school/peer risk** | | | | |
| Expelled from school | 1.65 | 0.50–5.39 | **6.51*** | 1.18–36.03 |
| Peers exhibiting social deviance | **2.66*** | 1.13–6.28 | 1.27 | 0.33–4.81 |
| Mostly older friends (2+ years) | 0.48 | 0.20–1.17 | **3.87*** | 1.03–14.56 |
| Victim of bullying | 1.06 | 0.45–2.48 | **9.80*** ** | 3.04–31.54 |
| **3. Parental mental illness, drug problems, legal involvement** | | | | |
| Par. mental health service use | **0.43#** | 0.18–1.02 | 1.43 | 0.47–4.37 |
| Parental drug service use | 1.76 | 0.63–4.95 | 1.06 | 0.28–4.05 |
| Parent with legal involvement | 1.78 | 0.75–4.24 | 1.26 | 0.30–5.25 |
| **4. Child's substance use** | | | | |
| Smoking/tobacco use | **3.61**** | 1.57–8.29 | 1.21 | 0.17–8.50 |
| Alcohol use | 1.29 | 0.49–3.36 | **5.98*** | 1.33–26.84 |
| Cannabis use | 0.84 | 0.27–2.55 | 0.78 | 0.22–2.76 |
| Other illicit drug use | 0.44 | 0.15–1.28 | 0.97 | 0.22–4.32 |
| **5. Child's psychiatric risk** | | | | |
| Anxiety disorders | 0.71 | 0.18–2.77 | 0.40 | 0.08–1.97 |
| Depressive disorders | 2.14 | 0.55–8.40 | 0.74 | 0.22–2.46 |
| Oppositional defiant disorder | **2.71#** | 0.99–7.42 | 0.87 | 0.26–2.93 |
| Conduct disorder | 1.68 | 0.54–5.22 | **15.77*** ** | 4.08–60.99 |
| ADHD | 0.89 | 0.23–3.49 | **10.11*** | 1.03–98.80 |
| Comorbidity: 2+ diagnoses | -- | -- | -- | -- |
| **6. Child's physical health** | | | | |
| Obesity | **0.44#** | 0.18–1.06 | 1.30 | 0.32–5.29 |
| Somatic complaints | **2.68*** | 1.20–5.99 | **3.58*** | 1.08–11.94 |
| Injury | 2.06 | 0.86–4.92 | 1.27 | 0.41–3.97 |
| Inflammation: CRP ≥3 mg/L | **2.46#** | 0.95–6.37 | 1.05 | 0.244.63 |

Significance: #p < .10 *p < .05 **p < .01 ***p < .001

[1] Not included in multivariate models.

<u>Note:</u> The weekly non-heroin opioid use versus heroin use comparison includes only those heroin users who had used non-heroin opioids weekly. This coding scheme was chosen to better capture progression from less severe to more severe opioid use. Heroin users who had never used non-heroin opioids weekly were not included. An underlying assumption of these comparisons is that those with weekly non-heroin opioid use progressed from any non-heroin opioid use and that those with heroin use progressed from weekly non-heroin opioid use. However, it is possible that participants were co-using (i.e., endorsed the two categories of use at the same point in time rather than at sequential points in time) or using in these categories in the "reverse temporal order."

© 2020 Shanahan L et al. *JAMA Pediatrics.*
